# Norepinephrine in Septic Shock: A Systematic Review and Meta-analysis

**DOI:** 10.5811/westjem.2020.10.47825

**Published:** 2021-02-16

**Authors:** Muhammad Azfar Ruslan, Kamarul Aryffin Baharuddin, Norhayati Mohd Noor, Mohd Boniami Yazid, Abu Yazid Md Noh, Andey Rahman

**Affiliations:** * Universiti Sains Malaysia, School of Medical Sciences, Department of Emergency Medicine, Kubang Kerian, Kelantan; † Hospital Tanah Merah, Department of Emergency Medicine, Kelantan, Malaysia; ‡ Hospital Universiti Sains Malaysia, Department of Emergency Medicine, Kelantan, Malaysia; § Universiti Sains Malaysia School of Medical Sciences, Department of Family Medicine, Kubang Kerian, Malaysia

## Abstract

**Introduction:**

Most experts recommend norepinephrine as the first-line agent in septic shock. Our objective was to determine the effectiveness and safety of norepinephrine in patients with septic shock.

**Methods:**

We searched the Cochrane Central Register of Controlled Trials and Epistemonikos, as well as MEDLINE from 1966 till August 2019. Screening of full texts, evaluation for eligibility, and data extraction were done by four independent reviewers. We estimated risk ratios (RR) and mean differences (MD) using a random-effects model with 95% confidence intervals (CI). The primary outcomes included the number of participants who achieved the target mean arterial pressure (MAP), time to achieve the target MAP, and number of participants with all-cause 28-day mortality. The secondary outcomes included the length of stay in the intensive care unit, length of hospital stay, incidence of arrhythmia and myocardial infarction, vasopressor-free days, and number of participants with all-cause 90-day mortality.

**Results:**

We identified 11 randomized controlled trials with a total of 4,803 participants. There was no difference in the number of participants who achieved the target MAP between those patients receiving norepinephrine and other vasopressors (RR 1.44; 95% CI, 0.32 to 6.54; P = 0.640; I^2^ = 94%; two trials, 116 participants). There was no significant difference in time to achieve the target MAP (MD −0.05; 95%, CI, −0.32 to 0.21; P = 0.690; I^2^ = 26%; two trials, 1763 participants) and all-cause 28-day mortality (RR 0.95; 95% CI, 0.89 to 1.02; P = 0.160; I^2^ = 0%; seven trials, 4,139 participants). Regarding the secondary outcome, norepinephrine may significantly reduce the incidence of arrhythmia as compared to other vasopressors (RR 0.64; 95% CI, 0.42 to 0.97; P = 0.030; I^2^ = 64%; six trials, 3974 participants). There was no difference in the incidence of myocardial infarction (RR 1.28; 95% CI, 0.79 to 2.09), vasopressor-free day (RR 0.46; 95% CI, −1.82 to 2.74) and all-cause 90-day mortality (RR 1.08; 95% CI, 0.96 to 1.21) between norepinephrine and vasopressors.

**Conclusion:**

In minimizing the occurrence of an arrhythmia, norepinephrine is superior to other vasopressors, making it safe to be used in septic shock. However, there was insufficient evidence concerning mortality and achievement of the target MAP outcomes.

## INTRODUCTION

Sepsis remains one of the significant causes of morbidity and mortality in critically ill patients worldwide despite the use of broad-spectrum antibiotics, advanced intensive care unit (ICU) management, and resuscitation strategies and protocols.^1^ More than 19 million sepsis cases and 5 million sepsis-related deaths are reported to occur annually, especially in low- and middle-income countries.^2^ According to the most recent report from the US Centers for Disease Control and Prevention, sepsis affects approximately 1.5 million people in the United States annually, resulting in 250,000 deaths, or one of every three hospital deaths.^3^ The incidence is increasing, mostly influenced by an aging population with multiple comorbidities, increased use of immunosuppressive therapy, and high-risk interventions.^4^

Definitions of sepsis and septic shock were revised in 2001 to incorporate the threshold values for organ damage. In 2016 there was a dramatic change in the definitions of sepsis and septic shock.^5^ Sepsis is now defined as life-threatening organ dysfunction caused by a dysregulated host response to infection. Organ dysfunction is characterized by an increase in the Sequential Organ Failure Assessment score of two points or more, which is associated with an in-hospital mortality greater than 10%. Patients with septic shock require vasopressor to maintain a mean arterial pressure (MAP) of 65 millimeters mercury (mm Hg) or greater and serum lactate level greater than 2 millimoles per liter (>18 milligrams per deciliter) in the absence of hypovolemia. This combination is associated with hospital mortality rates greater than 40%.^6^

The therapeutic goals in the management of sepsis are to improve tissue oxygenation and perfusion and to provide antimicrobial therapy with appropriate cover against the causative organism. The recent 2016 key recommendations include the following: 1) Intravenous (IV) antibiotics should be started within one hour of sepsis recognition; 2) patients with hypoperfusion should receive at least 30 milliliters per kilogram (mL/kg) of IV crystalloid within three hours and should be reassessed frequently; 3) for patients who require vasopressors, the initial target MAP should be 65 mm Hg; and 4) norepinephrine is the first choice for patients who need vasopressors, followed by vasopressin or epinephrine.^4^

The main pharmacological effect of norepinephrine is to increase organ perfusion by increasing vascular tone. Several studies comparing norepinephrine with dopamine favored the former in terms of overall improvements in oxygen delivery, organ perfusion, oxygen consumption, and less risk of arrhythmic effect.^7^ In this regard, norepinephrine is used primarily as a vasopressor to manage low systemic vascular resistance caused by vasodilation, which occurs in septic shock.^8^

A previous meta-analysis, which included trials up to January 2017, assessed outcomes such as mortality, oxygen delivery, oxygen consumption, cardiac index, heart ratio, MAP, mean pulmonary arterial pressure, central venous pressure, and systemic vascular resistance index.^9^ The current review evaluates other important outcomes and includes the latest trials, which may affect the findings of previous reviews. We performed a systematic review and meta-analysis to assess the effectiveness and safety of norepinephrine compared with other vasoactive agents and placebo in patients with septic shock. The primary outcomes included the number of participants who achieved the target MAP, time to achieve the target MAP, and all-cause 28-day mortality.

## METHODS

This systematic review was executed according to the protocol formerly published in the PROSPERO register. The methodology and reporting were constructed grounded on references from the Cochrane collaboration,^10^ and the preferred reporting items for systematic reviews and meta-analyses (PRISMA) statement.^11^ The appraisal was done according to the Grading of Recommendations, Assessment, Development and Evaluations (GRADE) approach.^12^

### Literature Search and Selection of Studies

We searched the Cochrane Central Register of Controlled Trials (Issue 8 of 12, August 2019), Epistemonikos, and MEDLINE (1966 to August 2019), using the text words “norepinephrine” and “septic shock” as well as Boolean operators such as “AND,” “OR,” truncation, and wildcards for variations in words. We restricted our search to English language publications. The reference list of identified randomized controlled trials (RCT) and articles were examined to find any unpublished or unidentified trials. Ongoing trials were also searched through the World Health Organization International Clinical Trials Registry Platform and ClinicalTrials.gov.

Four authors (MAR, MBY, AR, AY) selected the RCTs for inclusion, using these search strategies. Titles and abstracts were screened, and full-text copies of those that appeared relevant were obtained to determine whether they met the inclusion criteria. We contacted authors of trials, if necessary, to clarify study eligibility. Any disagreements between the review authors were resolved by discussion. We included RCTs comparing norepinephrine with other inotropes and vasopressors (dopamine, epinephrine, vasopressin, phenylephrine, terlipressin) or placebo administered intravenously. We excluded cross-over studies and those that did not report the outcomes of interest. The population of interest was comprised of patients, regardless of age, who were diagnosed with septic shock by clinicians.

### Data Extraction

Using a data extraction form, the review authors (MAR, MBY, AR, AY) independently extracted data on characteristics of the trials, participants’ characteristics, methodology, intervention, and outcomes. We attempted to contact the corresponding authors of trials if the information was missing or inadequately reported. Discordances at all stages were resolved through discussion.

### Outcome Measures

The primary outcomes of interest included the number of participants who achieved the target MAP, time to achieve the target MAP, and number of participants with all-cause 28-day mortality. Length of stay in the intensive care unit (ICU), length of hospital stay, incidence of arrhythmia, incidence of myocardial infarction, vasopressor-free days, and number of participants with all-cause 90-day mortality were considered as secondary outcomes.

### Assessment of Risk of Bias in Included Studies

We assessed the risk of bias based on random sequence generation, allocation concealment, blinding of participants and personnel, blinding of outcome assessors, completeness of outcome data, the selectivity of outcome reporting, and other bias. The Cochrane Collaboration tool for assessing the risk of bias was used to appraise the trials and is reported in the risk of bias table.^10^ We categorized risk of bias as low, high, or unclear. Any disagreements between the review authors were resolved by discussion.

### Primary Data Analysis

Review Manager (RevMan) version 5.3.5 (Nordic Cochrane Centre, Cochrane Collaboration, Copenhagen, Denmark) was used to perform the statistical analyses. For all the included trials with categorical outcomes, we calculated the risk ratios (RR) and 95% confidence intervals (CI). For numerical outcomes, the mean differences (MD), standardized mean differences (SMD), and 95% CIs were calculated. If data from two or more trials were included in an analysis of an outcome, we reported the results of the random-effects model. We pooled these measures in meta-analyses and drew forest plots.

The presence of heterogeneity was assessed via two steps. First, we evaluated obvious heterogeneity at face value by comparing populations, settings, interventions, and outcomes. Second, we assessed statistical heterogeneity using the I^2^ statistic.^10^ We used the following guide to interpret heterogeneity: 0–40% may not be important; 30–60% may represent moderate heterogeneity; 50–90% may represent substantial heterogeneity; and 75–100% would represent considerable heterogeneity.^10^ Our goal was to conduct subgroup analyses based on time intervals for the primary outcomes if there were adequate trials present for each group.

### Grading Quality of Evidence

We used the principles of the GRADE approach for evaluating the quality of evidence in this review.^12^,^13^ For each outcome, four review authors independently assessed the quality of evidence. This approach detailed four levels of quality – high, moderate, low, and very low – depending on the existence of the following five parameters: 1) risk of bias of included trials; 2) indirectness of evidence; 3) unexplained heterogeneity; 4) imprecision of results; and 5) study design. The GRADEpro GDT software (Evidence Prime, Inc., Hamilton, Ontario, Canada) was used to reflect the quality of evidence for each outcome.

## RESULTS

**Figure 1:**
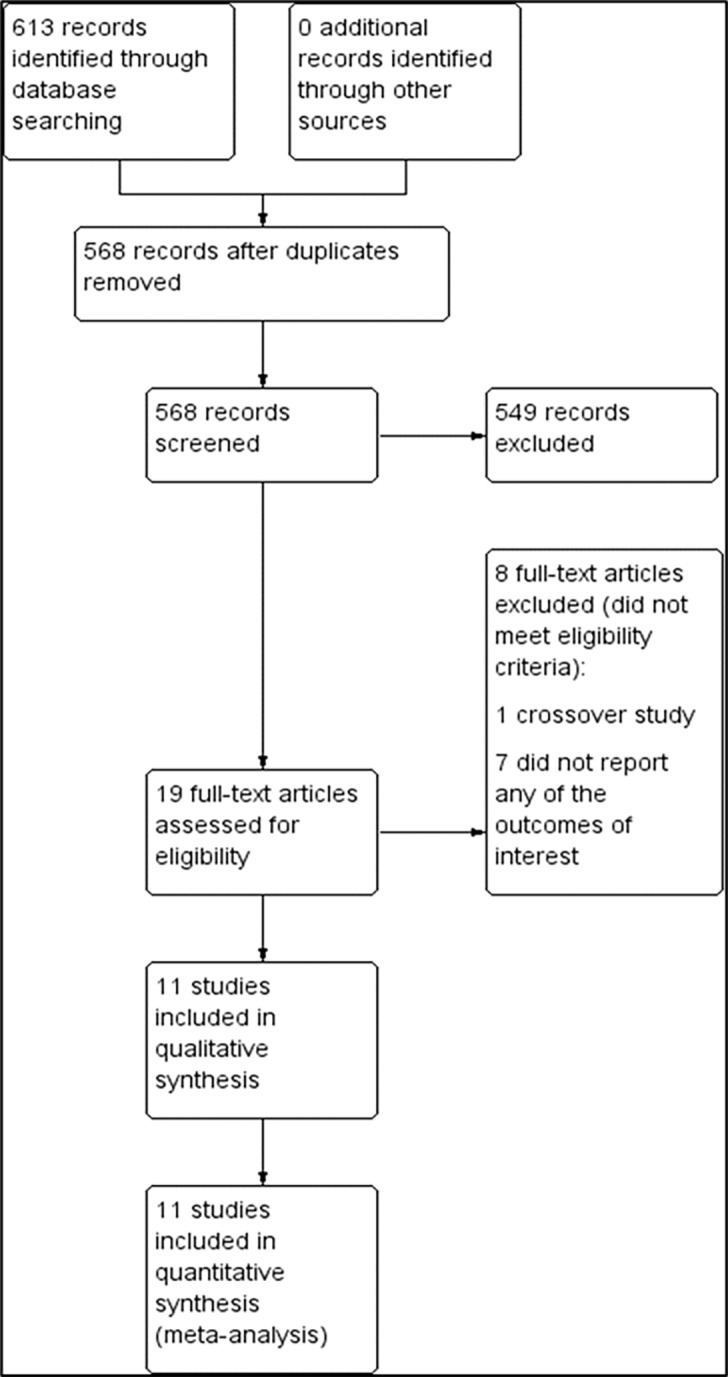
PRISMA flow chart of study selection for metanalysis of norepinephrine use in septic shock.

The preliminary search yielded 613 trials from the electronic databases according to the search strategy (Supplementary file, Figure 1). From these, 45 records were removed due to duplication. A total of 549 trials were excluded because their abstracts did not meet the inclusion criteria. We reviewed 19 full texts for eligibility, and excluded eight publications because one of them was a cross-over study^14^ and the remaining did not report the outcome of interest.^15^–^21^ Therefore, 11 trials with a total of 4,803 participants were included for systematic review and meta-analysis (Figure 1).^22^–^32^ Five trials were single-center studies,^23^,^27^,^28^,^30^,^31^ while six were multicenter studies.^22^,^24^–^26^,^32^

From a total of 4,803 participants, 2,368 were in the intervention group while 2,435 were in the control group. The study with the largest sample size had 1679 participants.^24^ All trials included participants older than 16 years of age. The diagnostic criteria for septic shock varied among the studies with the majority of trials using MAP less than 65–70 mm Hg after adequate fluid resuscitation as reference. All studies recruited participants from the ICU, except for one study that recruited from the emergency department.^31^ All trials used IV norepinephrine comparing with other agents such as dopamine, epinephrine, vasopressin, phenylephrine, terlipressin, and placebo, except for one study that used either norepinephrine alone or in combination with dobutamine.^22^ Three trials compared norepinephrine with dopamine,^24^,^27^,^30^ two trials compared norepinephrine with epinephrine,^22^,^29^ two trials compared norepinephrine with vasopressin,^25^,^32^ and two trials compared norepinephrine with terlipressin.^23^,^26^ Only one trial used placebo as a control.^31^ The dosage of drugs for both intervention and control groups varied among studies. Characteristics of the included trials are summarized in the Table. In this review, we formed two comparisons between 1) norepinephrine and vasopressors and 2) norepinephrine and placebo. We evaluated included studies as having low, high, or unclear risk of bias for each domain (Supplementary file, Figure 2). Generally, the risk of bias in each domain was reported to be low or unclear among the included studies. Risk of bias for individual studies is described in Supplementary file, Figure 3. All trials reported methods of randomization used. Eight trials used computer-generated randomization, and one trial used quasi-randomization based on odd or even day of the month.^30^ Two trials used block randomization.^23^,^26^ Three trials did not describe methods of allocation concealment used.^27^,^28^,^30^ All trials reported blinding of participants, personnel, and outcome assessor except in two trials, which were open-label studies.^23^,^30^ Nine trials carried out an intention-to-treat analysis.^22^–^26^,^29^–^32^ Ten trials reported the outcomes as specified in their protocols.^22^–^26^,^28^–^32^ Only one trial did not report any protocol.^27^

### Primary Outcomes

**Figure 2:**
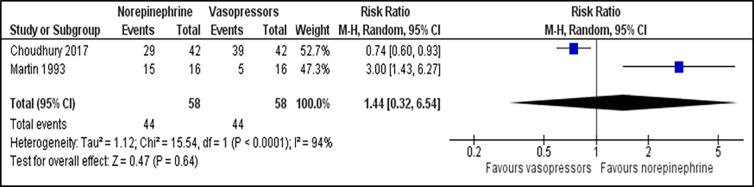
Comparison between norepinephrine and vasopressors for the outcome of the number of participants who achieved the target mean arterial pressure. *M-H*, Mantel-Haenszel method; *CI*, confidence interval.

**Figure 3:**
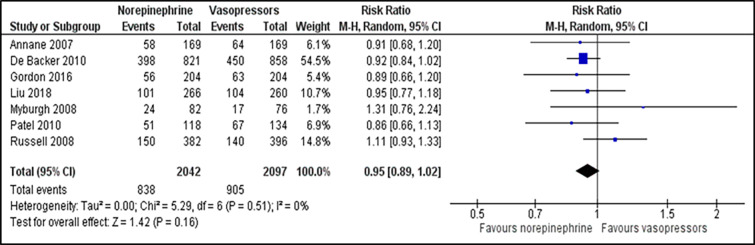
Comparison between norepinephrine and vasopressors for the outcome of the number of participants with all-cause 28-day mortality. *M-H*, Mantel-Haenszel method; *CI*, confidence interval.

For comparison between norepinephrine and vasopressors, the outcome for the number of participants who achieve the target MAP was reported in two trials.^23^,^27^ There was no significant difference in the number of participants who achieved the target MAP between those patients receiving norepinephrine and vasopressors (RR 1.44; 95% CI, 0.32 to 6.54; *P* = 0.640; I^2^ = 94%; two trials, 116 participants: low quality of evidence) (Figure 2). Two studies that reported time to achieve the target MAP 23,24 were analysed and revealed no significant difference between both groups (MD −0.05; 95% CI, −0.32 to 0.21; *P* = 0.690; I^2^ = 26%; two trials, 1,763 participants: high quality of evidence) (Supplementary file, Figure 4). For outcome of all-cause 28-day mortality, there was no significant difference between the norepinephrine and vasopressors groups (RR 0.95; 95% CI, 0.89 to 1.02; *P* = 0.160; I^2^ = 0%; seven trials, 4,139 participants: high quality of evidence) (Figure 3). For comparison between norepinephrine and placebo, the outcomes for the number of participants who achieved the target MAP and all-cause 28-day mortality were reported in one trial.^31^ Norepinephrine was superior to placebo in the number of participants who achieved the target MAP (RR 1.57;95% CI, 1.31 to 1.89; *P* < 0.001; one trial, 310 participants). There was no significant difference in the number of patients with all-cause 28-day-mortality between both groups (RR 0.71; 95% CI, 0.44 to 1.13; *P* = 0.150; one trial, 310 participants).

### Secondary Outcomes

**Figure 4:**
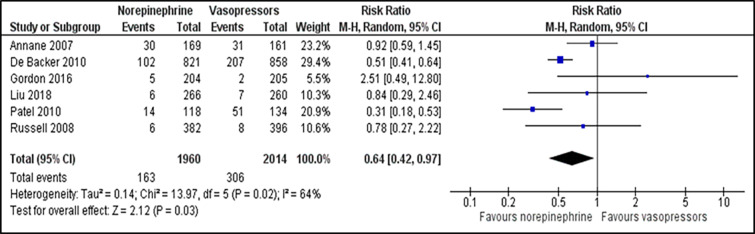
Comparison between norepinephrine and vasopressors for the outcome of the incidence of arrhythmia. *M-H*, Mantel-Haenszel method; *CI*, confidence interval.

We included all secondary outcomes in the meta-analysis except for length of ICU and hospital stay. Only one study reported length of hospital stay in mean number of days^30^ while the remaining reported in median. Norepinephrine was superior to other vasopressors in reducing the incidence of arrhythmia (RR 0.64; 95% CI, 0.42 to 0.97; *P* = 0.030; I^2^ = 64%; six trials, 3,974 participants: moderate quality of evidence) (Figure 4). There was a non-significant difference in incidence of myocardial infarction (RR 1.28; 95% CI, 0.79 to 2.09; *P* = 0.310; I^2^ = 0%; three trials, 2983 participants: high quality of evidence) (Supplementary file, Figure 5), vasopressor-free day (RR 0.46; 95% CI, 1.82 to 2.74; *P* = 0.690; I^2^ = 76%; two trials, 2,205 participants: moderate quality of evidence) (Supplementary file, Figure 6) and all-cause 90-day mortality (RR 1.08; 95% CI, 0.96 to 1.21; *P* = 0.440; I^2^ = 0%; three trials, 1,257 participants: high quality of evidence) (Supplementary file, Figure 7) between norepinephrine and vasopressors groups. For comparisons between norepinephrine and placebo, one trial^31^ reported the outcome of the incidence of arrhythmia, which revealed the significant advantage of norepinephrine over placebo (RR 0.55; 95% CI, 0.32 to 0.95; *P* = 0.030; one trial, 310 participants). Length of ICU stay was reported in eight trials, while the length of hospital stay was described in five trials.

## DISCUSSION

The 2018 Surviving Sepsis Campaign bundle introduced “hour-1 bundle,” which outlined five essential key elements to be considered within the first hour of recognition of sepsis patients in healthcare facilities. These elements include measuring lactate level, obtaining blood cultures, administering broad-spectrum antibiotics, and instituting 30 mL/kg IV crystalloid for the hypotensive patient, as well as administration of vasopressor to maintain a MAP of 65 mm Hg.^33^ It clearly shows the importance of early vasopressor use to maintain adequate tissue perfusion in septic shock patients, thus reducing mortality.

Using the GRADE approach, the quality of evidence among the measured outcomes ranged from moderate to high (Supplementary file, Table 1). Generally, the risk of bias in each domain for most trials was reported to be low or unclear. Only two trials showed a high risk of performance bias since both were open-label studies.^23^,^30^ All trials were classified as low risk in random sequence generation except for one study, which used randomization based on odd or even day of the month.^30^ The risk of reporting bias was present in one trial since there was no protocol provided.^27^

Evaluation of all three primary outcomes – namely, the number of participants who achieved the target MAP, time to achieve the target MAP, as well as all-cause 28-day mortality – revealed no significant difference between norepinephrine and other vasopressors. This is consistent with two previous meta-analyses, which had shown no mortality benefit of norepinephrine over other vasopressors such as dopamine, epinephrine, phenylephrine, and vasopressin.^9^,^34^ However, two reviews^35^,^36^ reported that norepinephrine was superior to dopamine for the outcome of 28-day mortality. Comparison between norepinephrine and placebo for the number of patients who achieved the target MAP showed significant benefit of norepinephrine over placebo. There was substantial heterogeneity (94%) in the outcome of the number of participants who achieved target MAP, possibly as a result of the differences in the definition of target MAP and the dosage of the drugs used among studies.

We chose two life-threatening adverse effects to be assessed as secondary outcomes: the incidence of cardiac arrhythmia; and myocardial infarction. Interestingly, the use of norepinephrine in septic shock is associated with a 36% and 45% reduction of incidence of arrhythmia, respectively, compared to other vasopressors and placebo. This may be explained by the beta-1 effect of norepinephrine, which increases cardiac contractility, thus increasing blood flow to the heart. A previous systematic review had also shown the superiority of norepinephrine over dopamine in reducing the risk of arrhythmia in septic shock patients.^36^ This review also indicates that the incidence of myocardial infarction was not different between the groups. Other adverse effects were not included as they were reported in variable ways. Additionally, we discovered no significant difference between both groups in terms of vasopressor-free day and all-cause 90-day mortality. We were unable to proceed with meta-analysis for outcomes of length of hospital and ICU stay since all trials reported the number of days in median rather than mean, except in one study.

This review has several strengths. We used a systematic search strategy and included only relevant RCTs. Four authors independently conducted trial screening and data extraction. We used Cochrane’s risk of bias tool to assess the quality of all studies and GRADE to evaluate the quality of evidence for important outcomes in this systematic review. We have updated this review with the addition of two recent trials.^26^,^31^ Two comparisons were made comparing norepinephrine and other vasopressors as well as with placebo, which had not been addressed in previous reviews. It is intended to demonstrate the strength of norepinephrine alone as opposed to other vasopressors or placebo, if any.

## LIMITATIONS

We acknowledge a few limitations in this review. Firstly, only 11 trials met the inclusion criteria. Therefore more clinical studies are required to confirm the findings. Pediatric age group was not included because of limited trials available. The largest contribution of the review is from a trial^24^ with the highest number of sample size (n = 1,679) and may have influenced the overall findings of this review. There were a few outcomes with moderate to substantial heterogeneity. These variable outcomes were most probably due to the variation of characteristics of populations and different dosages of vasopressors used in different studies, as well as different definitions of outcomes used among studies. We could not perform subgroup analysis due to inadequate information available. A funnel plot was not constructed due to insufficient studies contributing to each outcome. Our review included only English language publications. However, there is no evidence of a systematic bias in the use of language restrictions in systematic, review-based meta-analyses in conventional medicine.^37^ While we believe that all relevant trials have been included, we cannot rule out the possibility that additional trials may be unpublished or published in sources not accessible to our search.

## CONCLUSION

In summary, there is no sufficient evidence to prove that norepinephrine is superior to other vasopressors in terms of mortality and achievement of the target MAP. However, this meta-analysis demonstrated the superiority of norepinephrine in reducing the incidence of arrhythmia, making it the safest vasopressor to be used in septic shock. Larger RCTs should be conducted to prove the efficacy and safety of norepinephrine over other vasopressors. We recommend future trials to perform proper allocation concealment and blinding of participants and personnel to reduce the risk of bias. The trials should also emphasize outcomes related to parameters of end-organ perfusion, monitoring of the participants, and effects of norepinephrine on other internal organs.

## Supplementary Information



## Figures and Tables

**Table:** Summary of included studies.

Study ID	Setting	Country	Total randomized (n)	Mean age (years)	Intervention	Control	Primary outcome
Martin 1993	Single center, ICU	France	32	52.5	Norepinephrine (0.5 mcg/kg/min)	Norepinephrine (0.5 mcg/kg/min)	Systemic and regional haemodynamic achievement
Annane 2007	Multi-center, 19 ICU	France	330	63	Norepinephrine (0·2 mcg/kg/min min) ± dobutamine (5 mcg/kg/min)	Epinephrine (0·2 mcg/kg/min) ± placebo	28-day all-cause mortality
Morelli 2008	Single center, ICU	Italy	32	70	Norepinephrine	Phenylephrine	Study drugs requirement, systemic and regional haemodynamic achievement
Myburgh 2008	Multi-center, 4 ICU	Australia	280	59.9	Norepinephrine	Epinephrine	Achievement of MAP goal >24 h without vasopressors
Russell 2008	Multi-center, 27 centers	Canada, Australia, and USA	778	60.5	Norepinephrine (5 to 15 mcg/min)	Vasopressin (0.01 to 0.03 U/min)	28-day mortality of any cause
De Backer 2010	Multi-center, 8 ICU	Belgium, Austria, and Spain	1,679	67.5	Norepinephrine	Dopamine	Rate of death at 28 days
Patel 2010	Single center, ICU	USA	252	Not stated	Norepinephrine (maximum of 20 mcg/min)	Dopamine (maximum of 20 mcg/kg/min)	28-day all-cause mortality
Choudhury 2016	Single center, ICU	India	84	47.5	Norepinephrine (7.5 mcg/min to 60 mcg/min)	Terlipressin (1.3 to 5.2 mcg/min)	Achievement of MAP of >65 mm Hg and maintenance of the same for the initial 48 hours
Gordon 2016	Multi-center, 18 ICU	United Kingdom	409	66	Norepinephrine (maximum of 12mcg/min)	Vasopressin (maximum of 0.06 U/min)	Kidney failure-free days during the 28 days after randomization
Liu 2018	Multi-center, 21 ICU	China	617	61	Norepinephrine (4 to 30mcg/min)	Terlipressin (20 to 160 mcg/h)	28-day all-cause mortality
Permpikul 2019	Single-center, ED	Thailand	310	Not stated	Norepinephrine (0.05 mcg/kg/min)	Placebo (Dextrose 5% in water)	Shock control rate by 6 hours

*ICU*, intensive care unit; *mcg*, microgram; *kg*, kilogram; *min*, minute; *U*, unit; *h*, hour; *MAP*, mean arterial pressure; *ED*, emergency department.
