# Supplementary material for: Norepinephrine in Septic Shock: A Systematic Review and Meta-analysis
Source: West J Emerg Med. 2021 Feb 16;22(2):196–203. doi: 10.5811/westjem.2020.10.47825 (PMC7972398; doi:10.5811/westjem.2020.10.47825)
Supplement: Supplementary file 1 [file wjem-22-196-s001.docx]

**Supplementary Files**

CENTRAL

1. (norepinephrine OR noradrenaline):ti,ab,kw
2. (septic shock OR sepsis OR severe sepsis):ti,ab,kw
3. #1 AND #2

MEDLINE (PubMed)

1. (norepineprine[Title/Abstract] OR noradrenaline[Title/Abstract])
2. (septic shock[Title/Abstract] OR sepsis[Title/Abstract] OR severe sepsis[Title/Abstract])
3. #1 AND #2

Epistemonikos

1. (title:(norepinephrine) OR abstract:(norepinephrine)) AND (title:(septic shock) OR abstract:(septic shock))
2. (title:(noradrenaline) OR abstract:(noradrenaline)) AND (title:(septic shock) OR abstract:(septic shock))

Supplementary file. Figure 1. Search strategy

| Supplementary file. Table 1. Summary of findings for the main comparisons | | | | | | |
| --- | --- | --- | --- | --- | --- | --- |
| **Norepinephrine compared to vasopressors for septic shock** | | | | | | |
| **Patient or population**: septic shock  **Setting**: in-patient  **Intervention**: norepinephrine  **Comparison**: vasopressors | | | | | | |
| Outcomes | **Anticipated absolute effects^*^** (95% CI) | | Relative effect (95% CI) | № of participants  (studies) | Certainty of the evidence (GRADE) | Comments |
|  | **Risk with vasopressors** | **Risk with norepinephrine** |  |  |  |  |
| Number of participants who achieved target MAP | 759 per 1,000 | **1000 per 1,000** (243 to 1,000) | **RR 1.44** (0.32 to 6.54) | 116 (2 RCTs) | ⨁⨁◯◯ LOW ^a,b^ | Risk of bias- not serious, inconsistency-serious, indirectness- not serious, imprecision - serious |
| Time to achieve target MAP | The mean time to achieve target MAP was **0** | MD **0.05 lower** (0.32 lower to 0.21 higher) | - | 1763 (2 RCTs) | ⨁⨁⨁⨁ HIGH | Risk of bias- not serious, inconsistency- not serious, indirectness- not serious, imprecision – not serious |
| All-cause 28-day mortality | 432 per 1,000 | **410 per 1,000** (384 to 440) | **RR 0.95** (0.89 to 1.02) | 4139 (7 RCTs) | ⨁⨁⨁⨁ HIGH | Risk of bias- not serious, inconsistency- not serious, indirectness- not serious, imprecision – not serious |
| Incidence of arrhythmia | 152 per 1,000 | **97 per 1,000** (64 to 147) | **RR 0.64** (0.42 to 0.97) | 3974 (6 RCTs) | ⨁⨁⨁◯ MODERATE ^c^ | Risk of bias- not serious, inconsistency- serious, indirectness- not serious, imprecision – not serious |
| Incidence of myocardial infarction | 19 per 1,000 | **25 per 1,000** (15 to 40) | **RR 1.28** (0.79 to 2.09) | 2983 (3 RCTs) | ⨁⨁⨁⨁ HIGH | Risk of bias- not serious, inconsistency- not serious, indirectness- not serious, imprecision – not serious |
| Vasopressor-free days | The mean vasopressor-free days was **0** | MD **0.46 higher** (1.82 lower to 2.74 higher) | - | 2205 (2 RCTs) | ⨁⨁⨁◯ MODERATE ^d^ | Risk of bias- not serious, inconsistency- serious, indirectness- not serious, imprecision – not serious |
| All-cause 90-day mortality | 445 per 1,000 | **481 per 1,000** (427 to 538) | **RR 1.08** (0.96 to 1.21) | 1257 (3 RCTs) | ⨁⨁⨁⨁ HIGH | Risk of bias- not serious, inconsistency- not serious, indirectness- not serious, imprecision – not serious |
| ***The risk in the intervention group** (and its 95% confidence interval) is based on the assumed risk in the comparison group and the **relative effect** of the intervention (and its 95% CI).   **CI:** Confidence interval; **RR:** Risk ratio; **MD:** Mean difference | | | | | | |
| **GRADE Working Group grades of evidence** **High certainty:** We are very confident that the true effect lies close to that of the estimate of the effect **Moderate certainty:** We are moderately confident in the effect estimate: The true effect is likely to be close to the estimate of the effect, but there is a possibility that it is substantially different **Low certainty:** Our confidence in the effect estimate is limited: The true effect may be substantially different from the estimate of the effect **Very low certainty:** We have very little confidence in the effect estimate: The true effect is likely to be substantially different from the estimate of effect | | | | | | |

**Explanations**

a. Substantial heterogeneity (94%)

b. Small sample size (n=58)

c. Moderate heterogeneity (64%)

d. Substantial heterogeneity (76%)


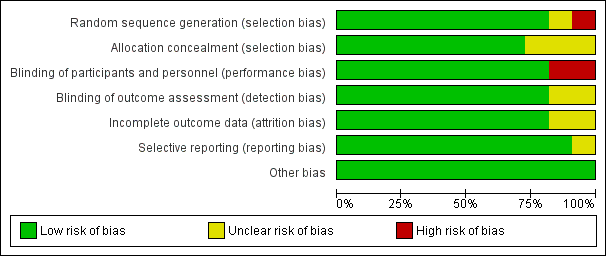


Supplementary file. Figure 2. ’Risk of bias’s graph: review authors’ judgements about each risk of bias item presented as percentages across all included studies.

**
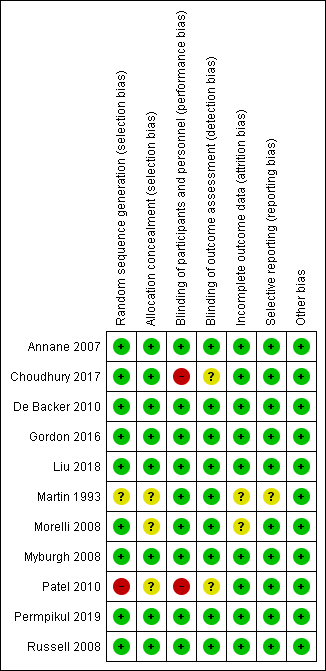
**

Supplementary file. Figure 3. ’Risk of bias’ summary: review authors’ judgements about each risk of bias item for each included study.


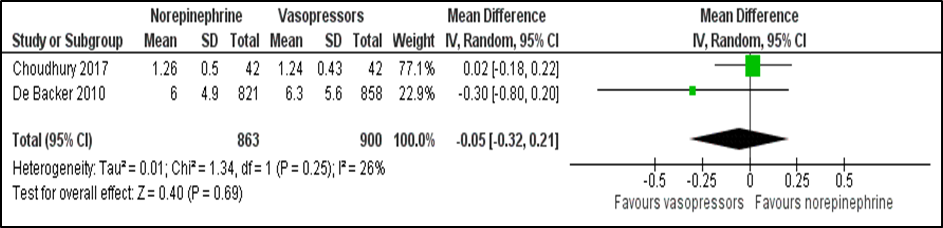


Supplementary file. Figure 4. Comparison between norepinephrine and vasopressors for the outcome of time to achieve target MAP.

**
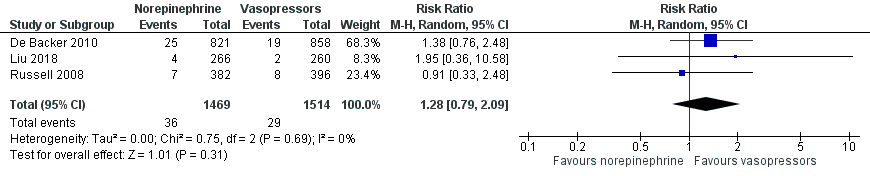
**

Supplementary file. Figure 5. Comparison between norepinephrine and vasopressors for the outcome of incidence of myocardial infarction

**
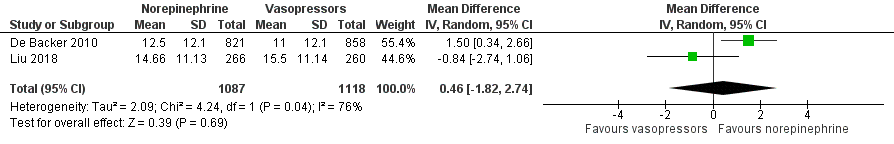
**

Supplementary file. Figure 6. Comparison between norepinephrine and vasopressors for the outcome of vasopressor-free day

**
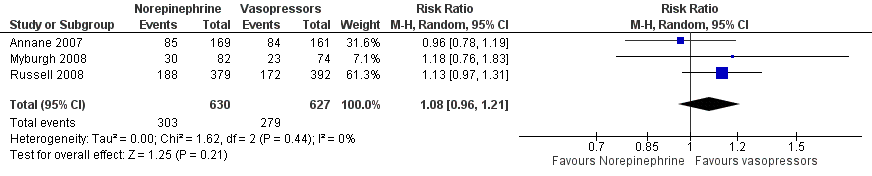
**

Supplementary file. Figure 7. Comparison between norepinephrine and vasopressors for the outcome of number of patients with all-cause 90-day mortality
